# Near-infrared autofluorescence of the parathyroid glands during thyroidectomy for the prevention of hypoparathyroidism: a prospective randomized clinical trial

**DOI:** 10.1007/s00423-022-02624-3

**Published:** 2022-07-29

**Authors:** Henning Wendelin Wolf, Norbert Runkel, Kathrin Limberger, Christian Andreas Nebiker

**Affiliations:** 1 Schwarzwald-Baar Klinikum Villingen-Schwenningen, Klinikstrasse 11, 78052 Villingen-Schwenningen, Germany; 2 Kantonsspital Aarau AG, Tellstrasse 25, 5001 Aarau, Switzerland; 3 AMEOS Spital Einsiedeln, Spitalstrasse 28, 8840 Einsiedeln, Switzerland

**Keywords:** Near-infrared autofluorescence, Hypoparathyroidism, Thyroid surgery, Hypocalcemia, Endocrine surgery

## Abstract

**Purpose:**

Postoperative hypoparathyroidism remains the most often complication in thyroid surgery. Near-infrared autofluorescence (NIR-AF) is a modality to identify parathyroid glands (PG) in vivo with high accuracy, but its use in daily routine surgery is unclear so far. In this randomized controlled trial, we evaluate the ability of NIR-AF to prevent postoperative hypoparathyroidism following total thyroidectomy.

**Methods:**

Patients undergoing total thyroidectomy were allocated in two groups with the use of NIR-AF in the intervention group or according to standard practice in the control group. The aim was to identify the PGs in an early most stage of the operation to prevent their devascularization or removal. Parathyroid hormone was measured pre- and postoperatively and on postoperative day (POD) 1. Serum calcium was measured on POD 1 and 2. Possible symptoms and calcium/calcitriol supplement were recorded.

**Results:**

A total of 60 patients were randomized, of whom 30 underwent NIR-AF-based PG identification. Hypoparathyroidism at skin closure occurred in 7 out of 30 patients using NIR-AF, respectively, in 14 out of 30 patients in the control group (*p*=0.058). There was no significant difference in serum calcium and parathyroid hormone levels between both groups. Likewise, NIR-AF could not detect PGs at a higher rate.

**Conclusion:**

The use of NIR-AF may help surgeons identify and preserve PGs but did not significantly reduce the incidence of postoperative hypoparathyroidism in this trial. Larger case series have to clarify whether there is a benefit in routine thyroidectomy.

**Trial registration number:**

DRKS00009242 (German Clinical Trial Register). Registration date: 03.09.2015

## Introduction

Injury, devascularization, or inadvertent removal of the parathyroid glands (PGs) during thyroidectomy can result in clinical hypoparathyroidism. As PG differs in shape, color, and location, even experienced surgeons perform inadvertent parathyroidectomy in up to 25% of thyroidectomies [1–4] and thus symptomatic hypocalcemia is the most common complication of thyroid surgery. Meta-analyses show a median incidence of transient and permanent hypocalcaemia post thyroidectomy of 27 and 1%, respectively [5]. Intraoperative frozen sections enable identification but carry the risk of partial or total damage to the glands.

Near-infrared (NIR) autofluorescence, introduced by Paras *et al.* in 2011, is a real-time, non-invasive intraoperative identification tool. PGs showed up to 8.5-fold higher fluorescence emission compared to surrounding tissue after exposure to NIR light [6]. Subsequent work emphasized the high sensitivity of this technique in identifying adenomatous as well as normal parathyroid glands and its usefulness in parathyroid gland surgery [7–10]. Recent studies describe decreasing rates of postoperative hypocalcemia after total thyroidectomy as well as higher PG detection rates [11].

This randomized controlled pilot trial analyzes the clinical capability of NIR autofluorescence to avoid hypoparathyroidism after total thyroidectomy for benign diseases. Furthermore, we discuss the feasibility of this method in daily surgical routine.

## Patients and methods

### Patients

**Fig. 1:**
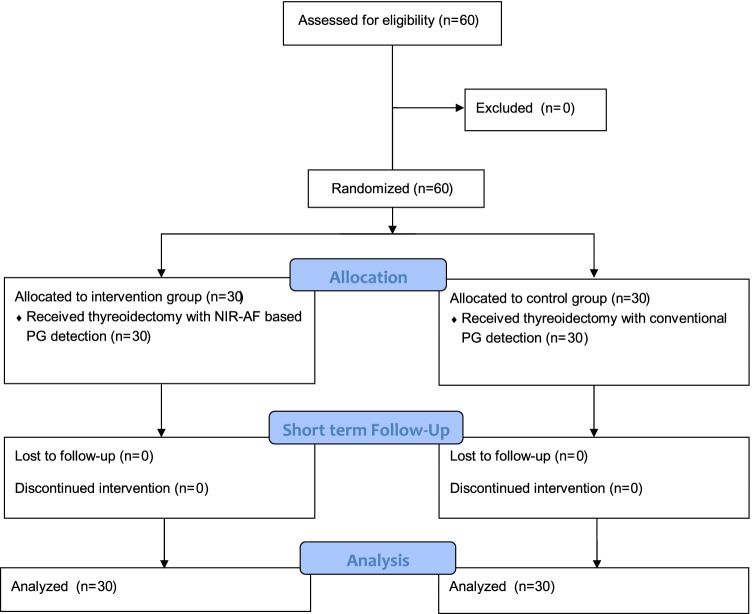
CONSORT 2010 flow diagram

Between August 2015 and October 2016, 60 consecutive patients undergoing total thyroidectomy for benign thyroid diseases in the Department of General Surgery in Villingen-Schwenningen (Germany) were enrolled in this study (Fig. 1). Exclusion criteria were previous thyroid or parathyroid operations, known primary or secondary hyperparathyroidism, and thyroid malignancies. The principal investigator performed the preoperative ultrasound and obtained informed consent from all patients. The study was registered with the German Clinical Trial Register (ID: DRKS00009242) and the approval of the local ethics committee was obtained. Preoperative software-based randomization (Randlist V1.2, DatInf GmbH, Tübingen, Germany) was used to allocate patients to the intervention group or control group. Due to the single-blinded study design, patients were not informed about their allocation.

Patients in the intervention group underwent total thyroidectomy with the use of NIR-AF, while in the control group, total thyroidectomy was performed without NIR-AF according to standard practice.

### Thyroid surgery

All operations were performed by six experienced surgeons (> 200 thyroid procedures per surgeon). Conversion to sternotomy was not necessary in any of the cases. Hemorrhage on the second postoperative day required revision surgery in one case.

### Technology

PG visualization based on autofluorescence does not require a tracer. NIR light was excited with a xenon light source whose light emitted through a filter has a wavelength of about 670–800 nm in “autofluorescence” mode (D-Light P, Karl Storz GmbH, Tuttlingen, Germany). The light was delivered to the tissue via a 10mm Hopkins 0° telescope while a high-resolution 3-chip camera unit (Tricam SL II, Karl Storz GmbH, Tuttlingen, Germany) was used to visualize the autofluorescence. This system had a CE clearance for autofluorescence and ICG-based imaging in laparoscopic or open surgery.

### Intraoperative measurements

**Fig. 2:**
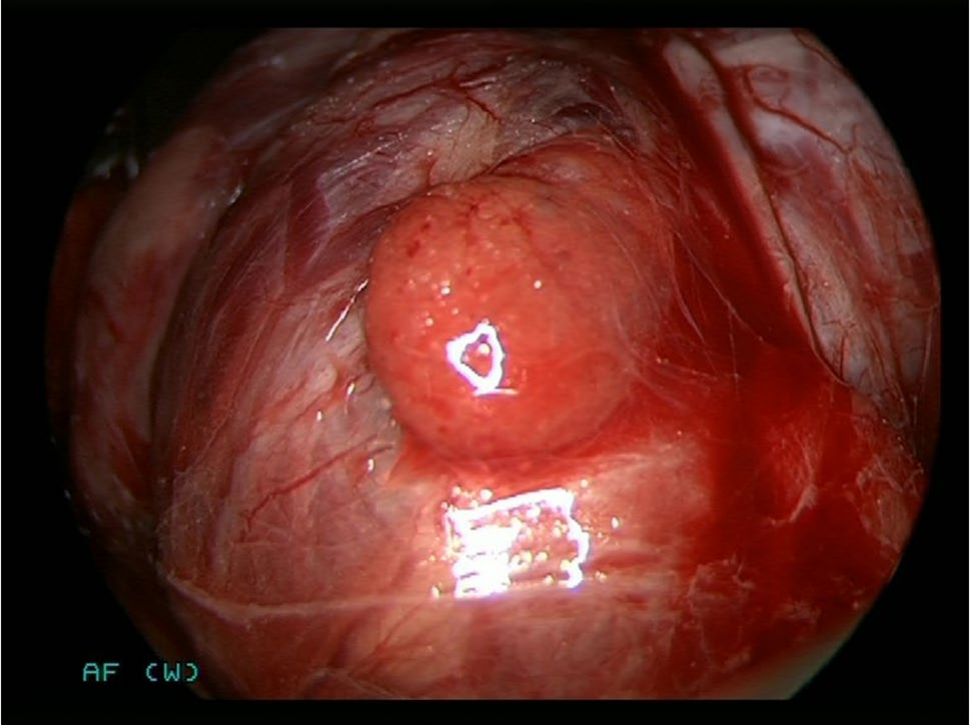
Lower right-sided parathyroid gland of a 71-year-old woman in white light.

**Fig. 3:**
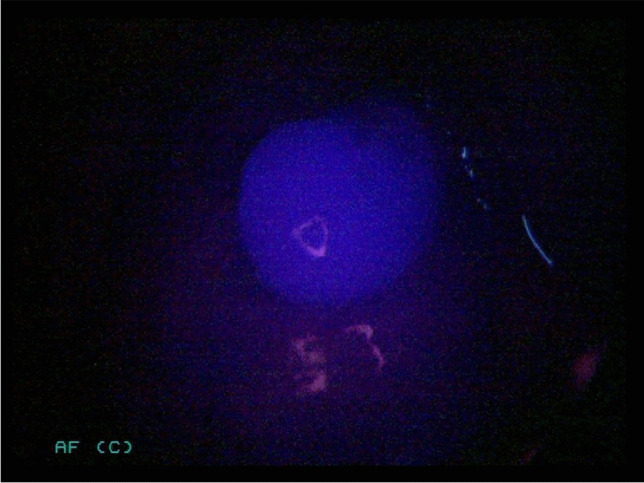
Same lower right-sided parathyroid gland of a 71-year-old woman illuminated with NIR light.

**Fig. 4:**
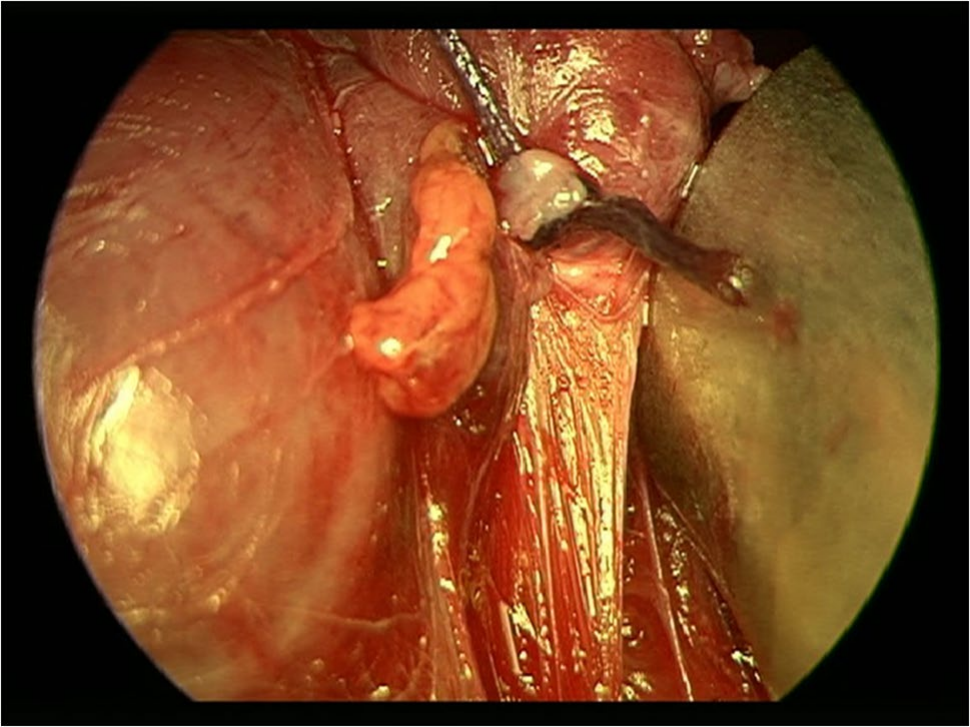
Upper left-sided parathyroid gland of a 71-year-old woman in white light.

**Fig. 5:**
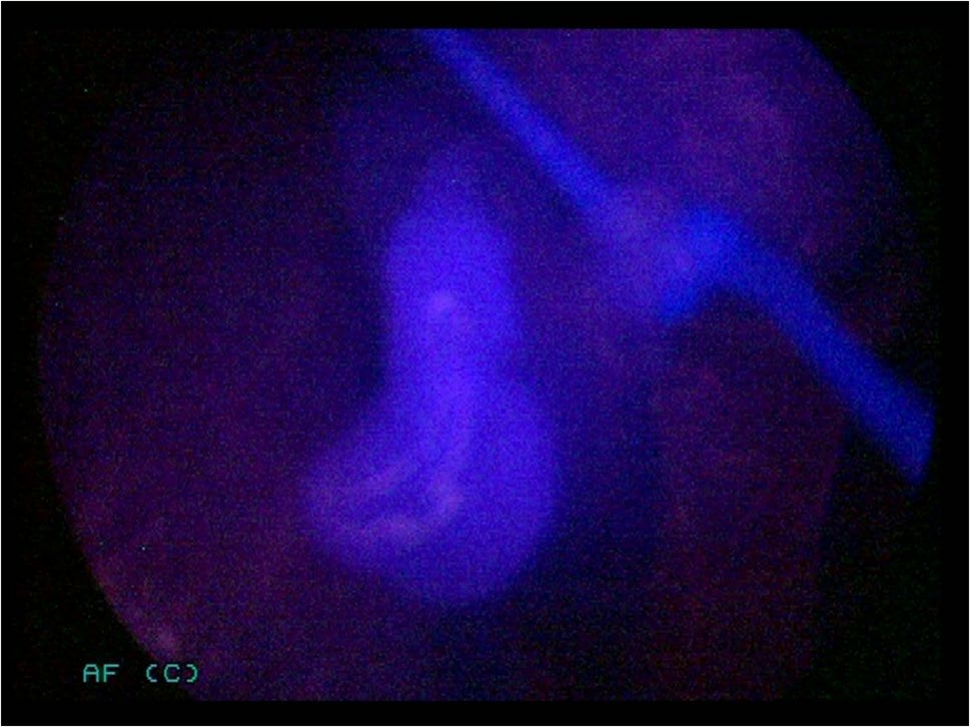
Same upper left-sided parathyroid gland of a 71-year-old woman illuminated with NIR light.

Before skin incision, a parathyroid hormone (PTH) measurement was carried out by intravenous blood sampling. Kocher’s incision was followed by mobilization of the larger thyroid lobe. The recurrent laryngeal nerve was identified and the superior and inferior PGs were identified visually by the surgeon. For patients in the intervention group, the lights in the OR were dimmed and NIR light was shone onto the visually recognized PGs. Based on previous experience, the distance between the light source/camera and parathyroid tissue was set to 2–3 cm to maximize autofluorescence (Figs. 2 and 3). Also based on experience of previous work (9), autofluorescence of the presumed PG was defined as “fluorescent” or “non-fluorescent” by the surgeon. A polyglactin suture (Ethicon Vicryl®) served as a reference as it emits fluorescence similar to parathyroid tissue (Figs. 4 and 5). If at least one PG was confirmed by NIR-AF, dissection of the thyroid lobe was completed. In the event that none of the visualized glands could be verified by NIR-AF, the search was continued along the thyroid capsule. Dissection of the opposite thyroid lobe and identification of the PGs was carried out using the same technique.

Following wound closure, a second PTH sample was taken. Documentation included the number of identified (visually and by autofluorescence) and autotransplanted PGs.

### Follow-up

PTH and calcium were measured on the morning of the first postoperative day (POD1). Calcium measurement was repeated on POD2. Patients were asked daily about symptoms of paresthesia or tetany. Any calcium and/or vitamin D substitution during hospitalization, as well as medication upon discharge and length of hospitalization, was documented. In case of hypocalcemia (absolute calcium levels lower than 2 mmol/L) or paresthesia, calcium was substituted orally. Calcitriol was additionally substituted for PTH values below 6 ng/L. If calcium was substituted during the hospital stay, a third lab test was performed on POD3. If necessary, calcium and/or calcitriol substitution was continued at discharge. Patients were routinely discharged on POD3. Hospitalization was prolonged by symptomatic hypocalcemia and/or the need for intravenous calcium substitution.

### Outcomes

The primary endpoint of this study was the incidence of hypoparathyroidism (PTH level < 12 ng/L) after skin closure and on the morning of POD 1. Calcium was measured without an albumin-based correction. Secondary endpoints were the number of PGs identified, the length of hospital stay (days between operation and discharge), the incidence of hypocalcemia-associated symptoms (paresthesia, tetany), and the need for oral substitution of calcium and calcitriol.

### Statistics

Because no effect of the technique was known when the study was designed, the number of cases was calculated based on the assumption that the incidence of hypoparathyroidism would be reduced by 25%. The reference incidence was based on our own incidence of postoperative hypoparathyroidism of 28% (own database of 746 consecutive thyroidectomies). Assuming a normal distribution, the *p* values were determined using a t-test or a Mann-Whitney-U test. The chi-squared test was used to examine the differences between categorical variables. The level of significance was taken to be *p*<0.05.

## Results

In total, 60 patients were allocated into two groups: 30 in the intervention group and 30 in the standard care group (Fig. 1). All patients were analyzed for the primary outcome as well as the short-term follow-up (at discharge). The groups did not differ in terms of age, gender, and thyroid volume (Table 1).

**Table 1:** Patients’ characteristics

	Intervention group (*n* = 30)	Control group (*n* = 30)	*p* value
**Gender**			
Male	9 (30%)	8 (27%)	0.77
Female	21 (70%)	22 (73%)	
**Age**			
Mean	57 (±12.7)	59 (±11.9)	0.51
Median	59	61	
Range	33-76	34-85	
**Indication**			
Compression symptoms	25 (83%)	30 (100%)	0.05
Graves’ disease	5 (17%)	0	
**Thyroid volume**			
Mean thyroid volume in mL	72 (±69)	70 (±60)	0.90
**Operations by surgeon**			
1	16	13	
2	1	9	
3	5	6	
4	4	1	
5	3	1	
6	1	1	
**Operation time**			
Mean operation time in min.	126 (±38)	122 (±48)	0.71
**PG identification**			
Mean PGs identified	3.03 (±0.75)	3.03 (±0.91)	1.0
PGs identified			
0	0 (0%)	1 (3%)	0.31
1	0 (0%)	1 (3%)	
2	8 (27%)	3 (10%)	
3	13 (43%)	16 (53%)	
4	9 (30%)	9 (30%)	
PGs autotransplanted	0.3 (±0.3)	0.43 (±0.6)	0.53
PGs inadvertently resected	1 (3%)	0	0.32

Mean values are followed by standard derivation (SD). Normal distributed values were analyzed using a two-sided T-test. The chi-squared or Fisher’s exact test was used to examine the differences between categorical variables

### Intraoperative findings

Operating time was slightly longer in the intervention group (+4 min; *p* = 0.71). The rate of patients with less than 2 identified PGs was lower in the intervention group (0 patients / %) than in the control group (2 patients, 7%). The rate of patients with 4 identified PGs was equal in both groups (9 patients, 30%). Histology reported one inadvertently resected PG in the intervention group, respectively none in the control group. Autotransplantation was less frequent in the intervention group (8 patients, 27%) than in the control group (11 patients, 37%; *p*=0.58).

### Postoperative findings

Absolute calcium values on POD1 and POD 2 did not differ significantly between both groups. The absolute and relative reduction between preoperative and POD 1 calcium values was slightly smaller in the intervention group compared with the control group. However, there was no difference in absolute and relative reduction between preoperative and POD2 calcium values in either group (0.25 ± 0.14 mmol/L in the intervention group, 0.29 ± 0.13 mmol/L in the control group; *p* = 0.31). There was also no significant difference in the incidence of postoperative hypocalcemia (calcium < 2.0 mmol/L) on POD 1 and 2 (*p* = 0.57; *p* = 1.0). The incidence of postoperative hypoparathyroidism at skin closure was less in the intervention group (7 patients, 23%) than in the control group (14 patients, 47%; *p* = 0.058). On POD1, 6 patients (20%) in the intervention group still showed a hypoparathyroidism, respectively 10 (33%) in the control group (*p* = 0.24). Overall, the incidence of postoperative hypoparathyroidism reduced by 23.6% at skin closure, and by 13.3% on POD1 using NIR-AF. Mean PTH levels after skin closure and on POD1 did not differ significantly in either group (Table 2). Neither was there a significant difference in the absolute and relative PTH values between the preoperative measurement and on POD1 in the two groups. Paresthesia was slightly lower in the intervention group (6 patients, 20%) than in the control group (8 patients, 27%; *p* = 0.76). There was no difference in the need for substitution of calcium and/or calcitriol at discharge (*p* = 0.84) or the length of hospital stay (*p* = 0.59).

**Table 2:** Laboratory and clinical results

	Intervention group	Control group	*p* value
	(n = 30)	(n = 30)	
Calcium absolute (mmol/L)			
PreOP	2.3 (±0.1)	2.33 (±0.11)	0.35
POD 1	2.09 (±0.14)	2.05 (±0.11)	0.22
POD 2	2.05 (±0.15)	2.04 (±0.10)	0.72
Calcium absolue reduction (mmol/L)			
PreOP to POD 1	0.21 (±0.14)	0.28 (±0.14)	0.07
PreOP to POD 2	0.25 (±0.14)	0.29 (±0.13)	0.31
Parathyroid hormone absolute (ng/L)			
PreOP	67.70 (±28.65)	74.12 (±35.79)	0.47
PostOP	28.85 (±17.01)	27.37 (±26.09)	0.36
POD 1	26.72 (±15.72)	24.75 (±17.30)	0.62
Parathyroid hormone absolute reduction (ng/L)			
PreOP to PostOP	38.9 (±33.86)	46.8 (±42.37)	0.28
PreOP to POD 1	41.0 (±27.9)	49.4 (±39.83)	0.48
Incidence of hypoparathyroidism			
Skin closure	7 (23%)	14 (47%)	0.058
POD 1	6 (20%)	10 (33%)	0.24
Incidence of hypocalcemia			
POD 1	7 (23%)	10 (33%)	0.57
POD 2	10 (33%)	10 (33%)	1
Symptomes postOP			
Paresthesia	6 (20%)	8 (27%)	0.76
Tetany	0	0	
Medication at discharge			
None	22 (73%)	20 (67%)	0.84
Calcium	2 (7%)	3 (10%)	
Calcium + calcitriol	6 (20%)	7 (23%)	
Hospitalization length (days)			
	3.2 (±0.52)	3.1 (±0.39)	0.59

Normal distributed calcium values were analyzed using a two-sided T-test. Parathyroid hormone values were analyzed using a Mann-Whitney-U test. Hypoparathyroidism is defined as PTH level < 12 ngl/L. Hypocalcemia defined as calcium level < 2.0 mmol/L. PTH and calcium levels and medication at discharge were analyzed using the Fisher exact test. The Chi-squared test was used to examine the differences between categorical variables. The average hospital stay after thyroidectomy was 3 days before the start of the study

## Discussion

Postoperative hypoparathyroidism remains an unpleasant complication for patients undergoing total thyroidectomy. Technical tools to assist the intraoperative identification of PGs are limited. Binocular loupes rely on the surgeon’s experience and do not provide additional imaging information [12], which can be acquired using fluorescence-based imaging techniques. Autofluorescence-guided intra-operative visualization of PGs is a groundbreaking technique shown to achieve excellent detection rates of both healthy and diseased PGs [6]. As no tracer is required, this real-time, repeatable method appears ideal for use in thyroid surgery to prevent inadvertent parathyroid injury or removal. In this randomized controlled trial, we examined the utility of NIR-AF in preventing postoperative hypoparathyroidism after total thyroidectomy.

### Parathyroid detection

This study did not show a higher mean detection rate of the PGs using NIR-AF but fewer patients with less than 2 detected PGs (Table 1). One single PG was detected by NIR-AF rather than visualization by the surgeon. In contrast, other authors demonstrated an increased number of PGs detected with NIR-AF [11, 13]. A known problem is the inability of NIR light to penetrate tissues beyond a depth of a few millimeters. As PGs are often covered by adipose tissue or blood, they elude stimulation by NIR light; however, Kahramangil et al. observed that 37–67% of all PGs could be recognized using NIR-AF before detection by the surgeons, even if they were covered by tissue [14]. Although a study from Cleveland, Ohio, demonstrated that once the overlying tissues are removed, NIR-AF identified PGs with a sensitivity of 98.5% [15], these glands are also likely to be identified by an experienced surgeon. Thus, this technique is more a real-time confirmation than a “navigation system” to the PGs. Although we were not able to detect an increased number of PGs by NIR-AF compared to visual identification alone, as surgeons we have all previously been confronted with the uncertainty as to whether a nodule corresponds to a PG and could have benefited from NIR-AF.

The technical system we used was image-based, and therefore, AF intensity was not quantified. As PGs display different AF intensities, influenced by external factors such as stray light, experience in interpreting the images is necessary. Spectroscopy-based systems may support less experienced surgeons by providing quantitative information, but these require physical contact with the presumed PG [16].

### Postoperative outcome

As shown in Table 2, we could not detect a significant difference in the occurrence of hypoparathyroidism at skin closure or POD1. Using NIR-AF, incidence reduced by 23.6% at skin closure, and by 13.3% on POD1. Postoperative parathyroid hormone and calcium blood levels were also not significantly different. Likewise, there was no difference in hypocalcemia-associated symptoms, medication at discharge, or length of hospital stay.

A key factor in preserving PG vascularization is careful vessel preparation along the thyroid capsule. Our intention was to identify PGs as early as possible during the operation. The Karl Storz® system used throughout the whole study requires partial dissection of the covering tissue to achieve sufficient AF intensity, as its ability to penetrate tissue is low. Especially in fatty conditions, this may increase the risk of accidental devascularization prior to identification. Laser-based systems are reported to have higher tissue penetrance [17, 18]. Benmiloud et al. reported a significant reduction in postoperative hypocalcemia using an integrated laser light-based system (Fluobeam® 800, Fluoptics, Grenoble, France). They started their trial just after the recruiting period of this study. With the mentioned system, significantly more PGs could be detected by NIR-AF [11]. An enhanced tissue penetrance may contribute to establishing NIR-AF as a real-time “mapping tool” for PGs in the early stages of thyroid surgery.

In both this and previous studies, we observed persistent AF activity in excised PGs. DiMarco et al. scanned thyroid specimens in 106 patients with an image-based system (Fluobeam® 800, Fluoptics, Grenoble, France) to detect inadvertently removed PGs but could find no difference in the number of PGs, early hypocalcemia or late hypoparathyroidism compared to 163 controls [18]. Therefore, the benefit of NIR-AF in the late operative phase remains unclear.

AF activity persists after PGs devascularization which compromises the ability of NIR-AF to serve as a decision tool as to whether to replant a PG. ICG angiography can facilitate this decision but requires an intravenous dye [19]. It can be surmised that NIF-AF-based identification together with ICG-based angiography has a high potential to preserve PGs. While a combined NIR-AF and ICG system with FDA and CE clearance already exists (Fluobeam LX®, Fluoptics, Grenoble, France), comparative studies are still lacking to prove its effectiveness.

In addition, one should not underestimate the learning curve both in visual and NIR-AF-based PG identification. We experienced a wide range of AF intensity depending on various conditions such as background lights, distance between the camera and tissue, and tissue penetrance. In our opinion, novice surgeons should undergo a training program before relying on NIR-AF.

### Limitations and future prospects

Due to the single-blinded study design, it is difficult to estimate how much the observer effect contributed to the results. In both the intervention and control groups, the participating surgeons reported a training effect in recognizing PGs. Thus, NIR could serve as a practice tool for both novice and experienced surgeons.

The participating six surgeons were not randomized to the two groups. Based on their experience (>30 thyroid procedures per year, >200 procedures in total) and a standardized operation technique, we assumed a low influence on the outcome.

The replantation rate of 27–37% in this study was high compared to 14.3% in 746 consecutive thyroidectomies prior to this trial. One explanation could be a forced search of the parathyroid glands by the surgeon (observer effect). Due to the system’s low tissue penetrance, this may have increased the risk of inadvertent devascularization.

The image system used was developed for ICG fluoroscopy but was modified with additional filters to be able to visualize NIR-AF. To ensure comparability of all study patients, we used the existing system and made no technical changes. This could be a disadvantage compared to systems originally designed for NIR-AF-based PG detection like the Fluobeam system (Fluoptics®, Grenoble, France). One advantage of the Storz® system is that it can also be used as a laparoscopic unit. Investment costs could thus be put into perspective.

The main limitation of this study is the small sample size based on the assumption of a 25% reduction in the incidence of postoperative hypoparathyroidism. A larger case series based on the effect demonstrated in our study could have substantially increased the power of this work.

The focus of our studies was on transient postoperative hypoparathyroidism. Therefore, no follow-up was performed. Since permanent hypoparathyroidism in particular represents a considerable burden for patients and is cost-intensive, a follow-up in a larger number of cases would be interesting.

## Conclusion

Near infrared-based identification of PGs during thyroidectomy could be a time and PG saving adjunct to conventional tissue preparation techniques. Presumably, we failed to significantly reduce the incidence of postoperative hypoparathyroidism due to the small sample size. Larger case series with optimized NIR parathyroid detection camera systems need to clarify whether there is a benefit in total thyroidectomy.

## Data Availability

Not applicable.
